# A Self-Healing PVA-Linked Phytic Acid Hydrogel-Based Electrolyte for High-Performance Flexible Supercapacitors

**DOI:** 10.3390/nano13030380

**Published:** 2023-01-17

**Authors:** Jing Zhao, Yuanqi Lu, Yuhua Liu, Lanxin Liu, Jinling Yin, Baozhi Sun, Guiling Wang, Yongquan Zhang

**Affiliations:** 1 Key Laboratory of Superlight Materials and Surface Technology of Ministry of Education, College of Materials Science and Chemical Engineering, Harbin Engineering University, Harbin 150001, China; 2 College of Power and Energy Engineering, Harbin Engineering University, Harbin 150001, China; 3 Jixi Quality Inspection and Testing Center of Graphite Product, Jixi 158100, China

**Keywords:** phytic acid, hydrogel electrolyte, self-healing, flexible supercapacitors

## Abstract

Flexible supercapacitors can be ideal flexible power sources for wearable electronics due to their ultra-high power density and high cycle life. In daily applications, wearable devices will inevitably cause damage or short circuit during bending, stretching, and compression. Therefore, it is necessary to develop proper energy storage devices to meet the requirements of various wearable electronic devices. Herein, Poly(vinyl alcohol) linked various content of phytic acid (PVA-PAx) hydrogels are synthesized with high transparency and high toughness by a one-step freeze-thaw method. The effects of different raw material ratios and agents on the ionic conductivity and mechanical properties of the hydrogel electrolyte are investigated. The PVA-PA_21%_ with 2 M H_2_SO_4_ solution (PVA-PA_21%_-2 M H_2_SO_4_) shows a high ionic conductivity of 62.75 mS cm^−1^. Based on this, flexible supercapacitors fabricated with PVA-PA_21%_-2 M H_2_SO_4_ hydrogel present a high specific capacitance at 1 A g^−1^ after bending at 90° (64.8 F g^−1^) and for 30 times (67.3 F g^−1^), respectively. Moreover, the device shows energy densities of 13.5 Wh kg^−1^ and 14.0 Wh kg^−1^ at a power density of 300 W kg^−1^ after bending at 90° and for 30 times during 10,000 cycles. It provides inspiration for the design and development of electrolytes for related energy electrochemical devices.

## 1. Introduction

In recent years, flexible energy storage systems have been developed with the increasing demand for portable and wearable electronic devices [1,2]. During the use of all kinds of wearable electronic devices, extrusion, stretching, collision, bending, and even puncture inevitably occur. Meanwhile, extremely harsh conditions due to climate differences will cause irreversible damage to all kinds of energy storage devices [3]. Therefore, it is urgent to develop novel energy storage devices with different functions to improve the flexibility, loss resistance, service life, and freezing resistance of electronic devices. These strategies will adapt to the challenges brought by different climate and environmental problems [4].

Supercapacitors have the advantages of ultra-high power density, long cycle life, and rapid charging and discharging [5]. Hydrogels are widely used as electrolytes for supercapacitors because of their great designability in structure and composition, as well as their anti-leakage properties compared with commonly used liquid electrolytes [6]. The combination of flexible electrodes and hydrogel electrolytes is needed to meet the requirements of the next generation of smart electronic devices [7].

Hydrogel-based supercapacitors with proper properties can be assembled through the combination of various materials such as PVA, PAA, and organic materials [8]. However, flexible hydrogel-based supercapacitors with excellent performance still have technical challenges. Nevertheless, how to accurately regulate various parameters is important to realize the interactive coordination of various additional properties of hydrogels [9]. The healing properties of self-healing hydrogels are conferred by dynamic covalent bonds or intermolecular attraction at both ends of the broken interface [10]. The former relies on a reversible covalent bond-breaking-contact-bonding mechanism. The latter includes the coordination of metals and ions, molecular recognition between host and guest, re-aggregation of long hydrophobic chains, and electrostatic attraction between oppositely charged ions and intermolecular induction [11]. Compared with conventional solid or liquid electrolytes, PVA-based hydrogel electrolytes are widely used to construct flexible supercapacitors due to their nontoxicity, safety, chemical stability, wide pH applicability, and relatively low cost. PVA is a linear structure polymer with a large number of hydrophilic hydroxyl groups on the surface. However, the poor water retention and unsatisfactory ionic conductivity of PVA limit practical applications [12]. To further explore the interacted bonding effect, we compared different hydrogels and the feasible physical self-healing methods, including hydrogen bonding, hydrophobic interaction, host-guest interaction, and Van Der Waals force (Table S1) [13,14,15,16,17,18,19,20].

In this work, phytic acid (PA) was introduced into PVA, and the PVA-PAx hydrogel electrolyte was prepared by the one-step gel-solvent method and the one-step freeze-thaw method. The formation of intermolecular hydrogen bonds improved the mechanical properties of PVA hydrogels. PA was considered as a gelator, which can improve the hydrogen bond interaction in PVA segmers, but it also reduces the PVA crystallization degree. Hence, the mechanical properties of the PVA-PA gels can be significantly enhanced. The PVA-PA_21%_ hydrogel possesses a large fracture stress (805 kPa). The PVA-PA_21%_-2 M H_2_SO_4_ hydrogel has an ultra-high ionic conductivity of 62.75 mS cm^−1^. Flexible supercapacitors based on PVA-PA_21%_-2 M H_2_SO_4_ hydrogel still have high specific capacitances at 1 A g^−1^ after bending 90° (64.8 F g^−1^) and for 30 times (67.3 F g^−1^). At the same time, the hydrogel electrolyte and activated carbon electrode were assembled to fabricate flexible supercapacitors with tensile and compressive functions. When the power density is 300 W kg^−1^, the energy densities after bending 90° and for 30 times are 13.5 Wh kg^−1^ and 14.0 Wh kg^−1^, respectively. Even at a current density of 4 A g^−1^, the capacity retention rate was 72.52% after 10,000 cycles.

## 2. Materials and Methods

### 2.1. Materials

Phytic acid (PA) was purchased from Shengjin Technology Co., Ltd. (Jingjiang, China). Potassium hydroxide (KOH) was obtained from Maclean Biochemical Technology Co., Ltd. (Shanghai, China). All chemicals were analytical reagents and were used without further purification.

### 2.2. Preparation of PVA-PAx Hydrogel Electrolytes

Firstly, 50 wt% phytic acid aqueous solution and deionized water were mixed according to the proportion in Table 1 (the total mass is constant). Then, the mixture was transferred to a round-bottomed flask after magnetic stirring evenly. Subsequently, 3 g of PVA was added, and the mixture was heated and stirred in an oil bath at 90 °C until the PVA was completely dissolved. Let stand for 10 min to allow air bubbles to escape, and then transfer the mixed solution to the mold. After cooling to room temperature, it was placed in a −18 °C refrigerator for 12 h, taken out, and thawed at room temperature to obtain PVA-PA_X_ (_X_ is the mass ratio of phytic acid) hydrogel. The name of each group of samples and the ratio of reactants are shown in Table 1 below:

Soak the prepared PVA-PAx hydrogel in 0.05 M H_2_SO_4_ solution for 6 h to achieve ion exchange equilibrium, and prepare a conductive hydrogel, which is recorded as PVA-PAx-0.05 M H_2_SO_4_. Hydrogels with different phytic acid contents and different electrolyte additions were prepared by the same method, which was denoted as PVA-PAx-Z M Y (where x is the content of phytic acid; Z is the electrolyte concentration (0.05, 0.2, 1, 2, 4); M is the abbreviation of mol L^−1^, and Y is one of the three electrolytes of H_2_SO_4_, KOH, and NaCl).

### 2.3. Characterization of the Synthesized Material

The infrared spectrometer of the Nicolet 6700 model was made by Thermo Fisher Scientific Corporation of the United States of America. The test spectral range was 4000 cm^−1^ to 450 cm^−1^ and the resolution was 4 cm^−1^. The instrument used in this paper is a TTR III X-ray diffractometer from Rigaku Corporation, Japan. The radiation source is Cu Kα (λ = 1.54056 Å) with a test range of 10–80°, and the scanning speed is 5° min^−1^. The morphology of the materials was tested by a scanning electron microscope (SEM, JEOL JSM-IT300LV).

### 2.4. Mechanical Properties Test of the PVA-PAx

The tensile testing machine was used to test the mechanical properties of the hydrogel, including tensile testing and compression testing. Among them, the tensile test sample is a strip with the size of 20 mm × 10 mm × 2 mm, and the tensile test is carried out at a rate of 10 mm min^−1^. The compression test sample is a cuboid sized 40 mm × 20 mm × 5 mm, with the compression test carried out at a rate of 2 mm min^−1^. The tensile stress and strain of the hydrogel electrolyte and the compressive stress when compressed to 80% were obtained through the test. The elongation/compression ratio is calculated by formula (1):

(1) $$\eta =\frac{l}{l_{0}}\;\times \;100\%$$

In the formula: η is the mechanical elongation/compression ratio; l_0_ is the length of the hydrogel electrolyte before tension/compression (mm); l is the length of the hydrogel electrolyte at tensile fracture/compression to 80% (mm).

### 2.5. Swelling Capability Test of PVA-PAx

The swelling ratio (SR) and equilibrium swelling ratio (SRmax) of the freeze-dried hydrogels soaked in water for a certain period of time were determined by the gravimetric method. The specific test method is as follows: the samples obtained after freeze-drying at −50 ℃ for 48 h are weighed and then soaked in water at room temperature, taken out at regular intervals, and the surface moisture is absorbed by filter paper and weighed until the soaking time becomes longer, and its quality is basically unchanged, that is, it reaches equilibrium. The swelling ratio is calculated by formula (2):

(2) $$\mathrm{SR}=\frac{W_{s}{\;-\;W}_{d}}{W_{d}}\;\times \;100\%$$

In this formula: SR is the swelling rate of the hydrogel; Ws is the mass of the hydrogel after swelling (g); Wd is the mass of the dry gel before swelling (g).

### 2.6. Ion Conductivity Test of PVA-PAx

The ionic conductivity was measured by the AC impedance method. The specific test method is as follows: soak the hydrogel in an aqueous electrolyte solution for a certain period of time to complete ion exchange, then cut it into a 20 mm × 10 mm × 5 mm cuboid, and clamp it with two polished titanium plates. In order to prevent the deformation of the hydrogel electrolyte during the fixing process, use a vernier caliper to re-measure its length, width, and height after fixing, and then use the AC impedance test (EIS) to obtain its body resistance Rb (Ω). The ionic conductivity σ (mS cm^−1^) is calculated by formula (3):

(3) $$\sigma =\frac{d}{A\cdot R_{b}}{\;\times \;10}^{3}$$

where σ is the ionic conductivity of the hydrogel electrolyte (mS cm^−1^); d is the distance between the two titanium plates (cm); A is the cross-sectional area of the electrolyte sandwiched by the titanium plates (cm^2^).

### 2.7. Assembly of Symmetric Supercapacitors

143 mg polyvinylidene fluoride (PVDF) solution (7 wt%), 80 mg activated carbon and 10 mg Super P were sequentially added into a 10 mL crucible under magnetic stirring, N-methylpyrrolidone (NMP) was added dropwise to make the slurry evenly mixed, and magnetic stirring was performed for 4 h. Then the slurry was coated on the surface of the carbon cloth that had been ultrasonically treated with acetone, ethanol, and deionized water. Finally, the carbon cloth loaded with activated carbon was transferred to a vacuum oven at 60 °C overnight to obtain the electrode of the supercapacitor. The mass of the electrode is 2 mg. The electrochemical performances were tested with a multi-channel electrochemical workstation (French-made VMP3 Bio-Logic).

## 3. Results and Discussions

### 3.1. Ionic Conductivity of PVA-PAx Hydrogels

**Figure 1:**
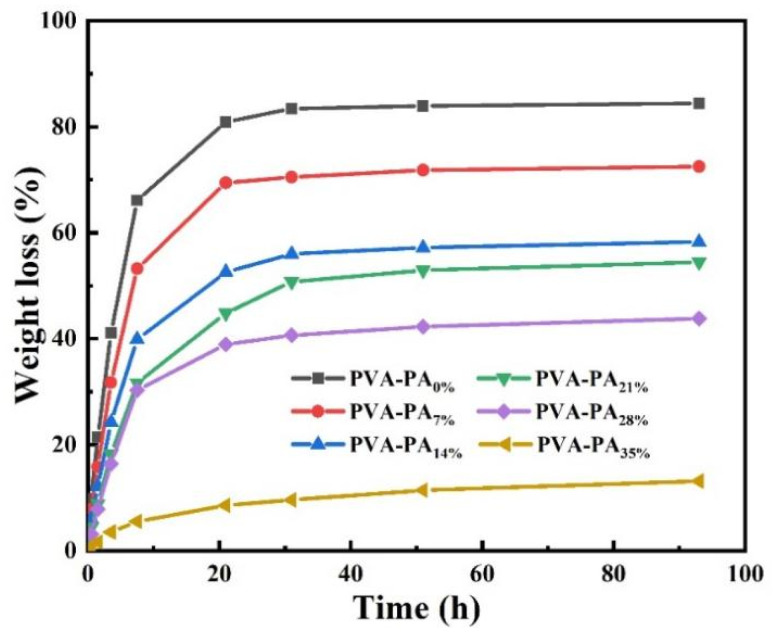
Weight loss of PVA-PAX hydrogels with different PA contents (20 ℃, humidity 15%).

Before exploring the ionic conductivity of hydrogels, the dehydration of hydrogels was first explored. Typically, water loss will lead to reduced hydrogel flexibility, reduced ionic conductivity, and device deformation [21]. The effect of phytic acid addition on the gel weight loss rate was explored through the hydrogen bond between phytic acid and polyvinyl alcohol (Figure 1). As expected, the pure PVA hydrogel lost water rapidly, and its weight loss rate reached 66% when exposed to air for 7.5 h. The weight loss rates were 53%, 40%, 32%, 30%, and 6%, respectively, showing an obvious decreasing trend. After 31 h, the pure PVA hydrogel and PVA-PAx hydrogel reached the weight loss plateau successively, and the weight loss rate was 84%, 72%, 58%, 54%, 43%, and 13%, which also showed a clear decreasing trend. This may be related to the formation of hydrogen bonds between phytic acid and PVA in water, reducing water evaporation. However, the content of deionized water in the system decreases with the increase of phytic acid addition and phytic acid does not volatilize. Hence, the weight loss rate decreases with the increase of phytic acid addition [22].

**Figure 2:**
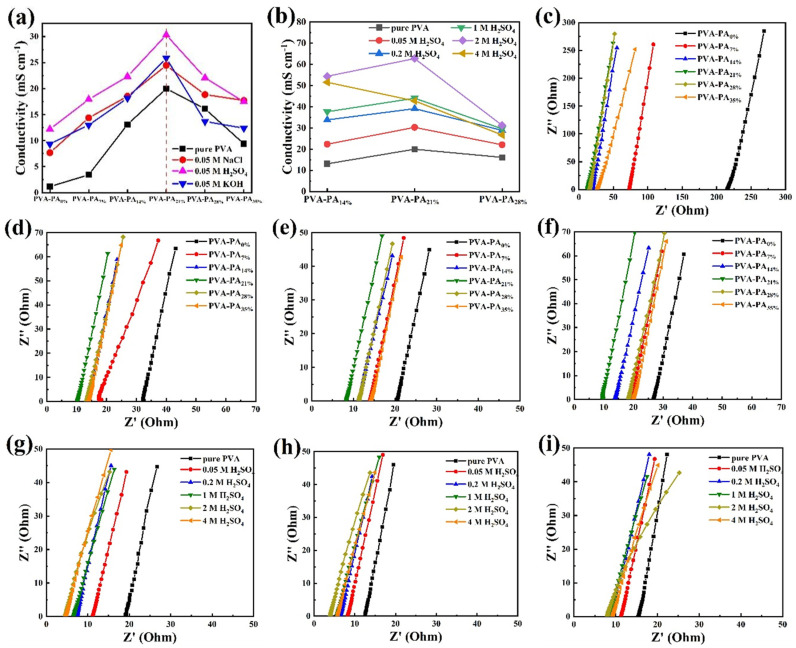
PVA-PAx-Z M Y hydrogel electrolyte ionic conductivity (**a**) without and with 0.05 M different electrolytes, (**b**) with different concentrations of H_2_SO_4_. Impedance tests of PVA-PAx-Z M Y hydrogel electrolyte added (**c**) without addition, with (**d**) NaCl, (**e**) H_2_SO_4,_ and (**f**) KOH. Impedance tests of (**g**) PVA-PA_14%_, (**h**) PVA-PA_21%_, (**i**) PVA-PA_28%_ with different concentrations of H_2_SO_4_.

Subsequently, the ionic conductivity of the hydrogel electrolytes prepared with different raw material ratios and different electrolyte additions was tested by the AC impedance method (as shown in Figure 2c–i). The specific numerical values of ionic conductivity are shown in Figure 2a,b. Figure 2a shows the ionic conductivity of the hydrogel electrolytes of PVA-PAx hydrogels with different PA content without adding electrolytes and adding 0.05 mol L^−1^ of H_2_SO_4_, KOH, and NaCl, respectively. The hydrogel electrolyte is also conductive without adding electrolyte, which may be related to the large number of phosphate groups in phytic acid that can ionize hydrogen ions in the hydrogel. With the increase of phytic acid content, the conductivity first increases and then decreases [23]. When the phytic acid content is 21 wt%, the ionic conductivity of PVA-PA_21%_ hydrogel electrolyte is the highest, which is 19.99 mS cm^−1^. This may be related to the ionic conductivity of PVA-PA_21%_ hydrogel electrolyte and the water content in the hydrogel. The degree of ionization of phytic acid is greater with a low phytic acid concentration [24]. When the water in the hydrogel decreased, the degree of ionization is lower due to the formation of hydrogen bonds between water and phytic acid. Hence, the ionic conductivity decreased with the decreasing water content [25]. The trend of conductivity when adding 0.05 mol L^−1^ H_2_SO_4_, KOH, and NaCl is basically the same as that without adding electrolyte, which increased first and then decreased. When adding 0.05 mol L^−1^ H_2_SO_4_ to hydrogel PVA-PA_21%_, the ionic conductivity is the largest (30.30 mS cm^−1^). The additions of 0.05 mol L^−1^ KOH and NaCl result in a similar conductivity, 25.88 and 24.44 mS cm^−1^.

Then, the effect of different concentrations of H_2_SO_4_ on the conductivity of the hydrogel electrolyte was explored by increasing the concentration of H_2_SO_4_ (Figure 2b). When the concentration of H_2_SO_4_ is between 0–2 mol L^−1^, the ionic conductivity of the hydrogel electrolyte with the same PA content increases continuously with the increase of H_2_SO_4_ concentration. When the H_2_SO_4_ concentration is the same, the ionic conductivity of different hydrogel electrolytes increases first and then decreases with the increase of PA content. Among them, the ionic conductivity of PVA-PA_21%_-2 M H_2_SO_4_ hydrogel electrolyte is the largest of 62.75 mS cm^−1^, which was used as the follow-up research material. However, the ionic conductivity decreased when the concentration of H_2_SO_4_ increased to 4 mol L^−1^. From the appearance, the hydrogel electrolyte was soft and difficult to maintain the original shape, which may be related to the existence of phytic acid. It presented good acid resistance, but phytic acid will be hydrolyzed into inositol and phosphate ester in a strong acid environment [26].

### 3.2. Mechanical Properties of PVA-PAx Hydrogels

**Figure 3:**
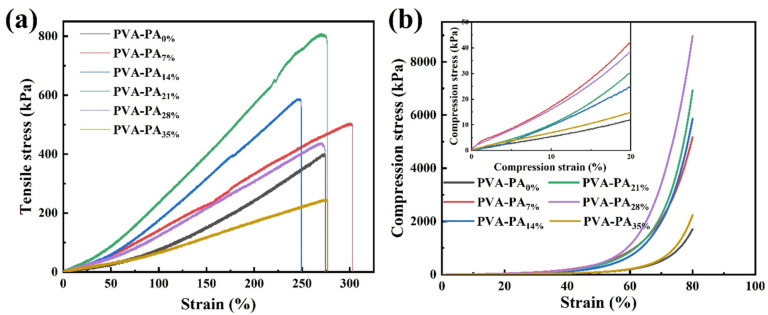
(**a**) Tensile stress-strain curve of PVA-PAX hydrogel. (**b**) Compressive stress-strain curve.

To explore the mechanical properties of the hydrogel electrolyte, the tensile/compressive stress-strain tests on the PVA-PAx hydrogel electrolyte were further performed (Figure 3). The pure PVA hydrogel as shown in Figure 3a exhibits satisfactory mechanical properties with an ultimate stress of 404.4 kPa and a fracture strain of 274.8%. With the increase of phytic acid addition, the fracture strains were maintained between 249% and 303%. When the phytic acid addition amount was 14 wt%, the fracture strain was the smallest at 249.2%. When the phytic acid addition amount was 7 wt%, the fracture strain reached the maximum of 302.8%. The ultimate stress increased first and then decreased with the increase of the content of phytic acid. The hydrogel PVA-PA_21%_ exhibited the highest ultimate stress of 805 kPa, which was twice that of pure PVA. Moreover, the PVA-PA_21%_ hydrogel exhibited a high strain of 276.5% at 805 kPa.

The PVA-PAx hydrogel has excellent resistance to compression (Figure 3b). The pure PVA hydrogel exhibited a compressive stress of 1711.6 kPa at 80% strain. With the increase of phytic acid addition, its compressive stress at 80% strain first increased and then decreased. When the phytic acid content was 28 wt%, the maximum compressive stress reached 8980.2 kPa. The PVA-PA_21%_ hydrogel also exhibited a compressive stress of 6937.8 kPa, which was four times that of the pure PVA hydrogel.

Therefore, the PVA-PA_21%_ hydrogel added with 2 mol L^−1^ H_2_SO_4_ presented excellent ionic conductivity (62.75 mS cm^−1^), high tensile strain (276.5%), satisfactory limit stress (805 kPa) and excellent compressive resistance (compressive stress of 6937.8 kPa at 80% compressive strain).

### 3.3. Structure and Morphology Characterizations

**Figure 4:**
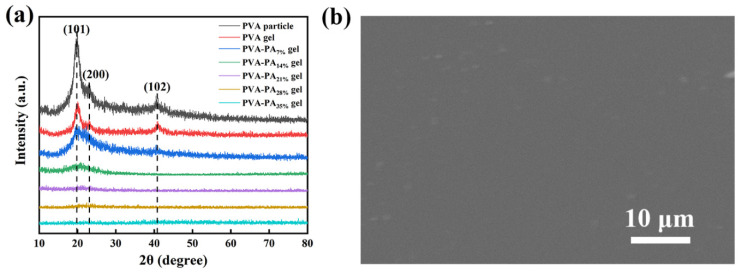
(**a**) XRD patterns of PVA-PAx hydrogels with different PA content. (**b**) SEM images of PVA-PA_21%_ hydrogels.

The effect of different PA additions on PVA crystallization was explored by XRD (Figure 4a). In the XRD spectrum of the PVA, there were obvious diffraction peaks at 2θ = 19.80°, 23.10°, and 40.80°, which are matched with the (101), (200), and (102) crystal planes of PVA, respectively. The intermolecular hydrogen bond of PVA was crucial to the formation of polymer crystallization at low temperatures. Therefore, the PVA film after one-step freeze-thaw did not reach the crystallization peak intensity of PVA. Phytic acid contains a large number of phosphoric acid groups, which can also form intermolecular hydrogen bonds with PVA. Therefore, the addition of phytic acid will affect the probability of the formation of intermolecular hydrogen bonds in PVA, thereby affecting the formation of polymer crystals by PVA at low temperatures. With the increase in the amount of phytic acid added, the crystallization peak intensity of the film gradually decreased until it disappeared, and the film at this time showed an amorphous state. Figure 4b showed the SEM image of PVA-PA_21%_ hydrogel. The prepared hydrogel has a uniform surface with wrinkles and micropores, which may promote electrolyte uptake and ion conduction [27].

**Figure 5:**
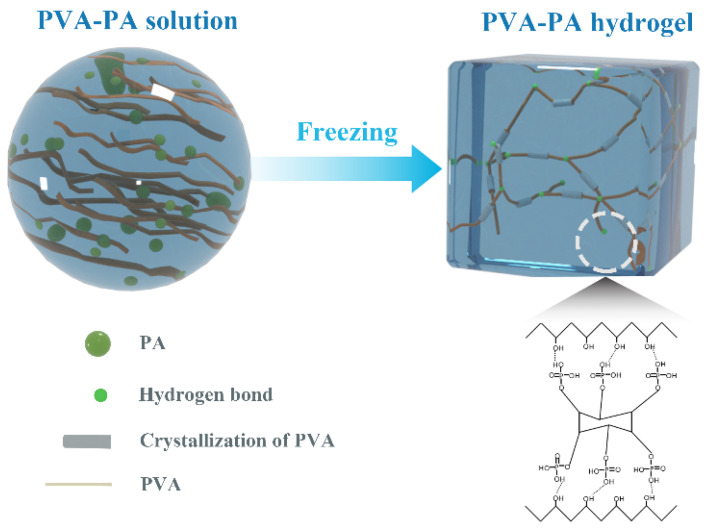
Synthesis mechanism diagram of PVA-PAx hydrogel.

The ion-conducting hydrogels were prepared by a one-step sol-gel synthesis of PVA and PA (Figure 5). The hydrogel utilized the hydroxyl groups in both PVA and PA, which can form intermolecular hydrogen bonds. Moreover, the crystallization behavior of PVA under freezing conditions was explored to prepare an ionic conductive hydrogel with strong mechanical properties and optical transparency. In the PVA-PAx hydrogel, the role of PA mainly included: (1) PA contains a large number of phosphoric acid groups, which can ionize a large number of hydrogen ions in the hydrogel, thereby increasing the ionic conductivity of the hydrogel [28]; (2) PA contains a large number of hydroxyl groups, which can form intermolecular hydrogen bonds with the hydroxyl groups in PVA so that the PVA hydrogel can be reconstructed in structure. At the same time, the formation of intermolecular hydrogen bonds also provides hydrogels with different physical cross-linking points [29]; (3) the introduction of PA disrupts the original morphology and order of PVA crystallization under freezing conditions, reducing the formation of intermolecular hydrogen bonds by PVA. Thus, the crystallization of PVA was reduced and the optical transparency of the conductive hydrogel increased [30].

**Figure 6:**
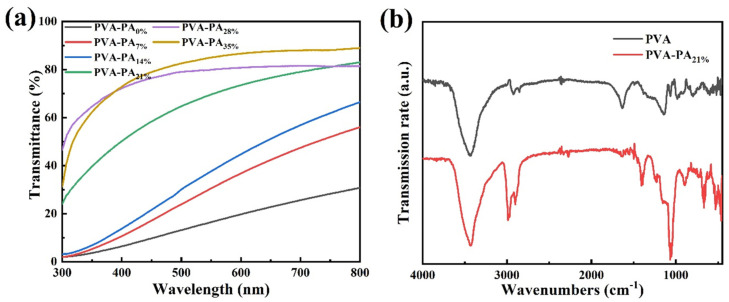
(**a**) UV-vis spectra of PVA-PAx hydrogels with different PA contents. (**b**) FT-IR spectra of pure PVA and PVA-PA_21%_.

The transmittance spectra of PVA-PAx hydrogels with different PA contents in the UV-Vis region are shown in Figure 6a. PVA-PAx hydrogel has better transparency than pure PVA hydrogel. At 700 nm wavelength, the transmittance of pure PVA hydrogel is only 25.6%, while the transmittance of hydrogel containing 35 wt% PA is as high as 88%. Such high transparency was caused by the introduction of PA, which disrupted the original morphology and order of PVA crystallization under freezing conditions. These results will reduce the formation of intermolecular hydrogen bonds and improve the optical transparency of the conductive hydrogel. The transmittance gradually increased with the increase of PA content, and the hydrogel with PA content of 21 wt% also showed a good transmittance of 79% at 700 nm wavelength.

The FT-IR spectra of pure PVA and PVA-PA_21%_ hydrogel are shown in Figure 6b. The absorption peak of pure PVA hydrogel at 3437 cm^−1^ was attributed to the stretching vibration peak of the associated hydroxyl group of the PVA molecule. While in the PVA-PA_21%_ hydrogel, the absorption peak moved to 3430 cm^−1^ due to the formation of intermolecular hydrogen bonds between PVA and PA, thus shortening the length of hydrogen bonds and leading to a shift in the frequency of the hydroxyl group to lower wavenumbers. The absorption peaks of PVA-PA_21%_ hydrogel at 2988 cm^−1^ and 2899 cm^−1^ corresponded to the asymmetric and symmetrical stretching vibration peaks of CH_2_ in the PVA molecule [31]. The peak at 1638 cm^−1^ was assigned to the deformation vibration peak of the hydroxyl group. Compared with the peak of pure PVA hydrogel at 1624 cm^−1^, the peak becomes smaller and the frequency shifted to a lower wavenumber. A special peak of PVA-PA_21%_ hydrogel appeared at 1395 cm^−1^, which may be the CH_2_ bending vibration peak of PVA. The characteristic peak that appeared at 1056 cm^−1^ was attributed to the characteristic peak of the PO_4_^3-^ group. The characteristic peak of the cluster in FT-IR was shifted to a higher wavenumber of 960 cm^−1^, indicating the formation of hydrogen bonds between phytic acid and PVA [32].

### 3.4. Electrochemical Performance Test of the Assembled Supercapacitor

A flexible symmetric supercapacitor was fabricated with PVA-PA_21%_-2 M H_2_SO_4_ hydrogel electrolyte and activated carbon electrodes, the electrochemical performances were tested to further investigate the practical applications.

The voltage range of flexible hydrogel supercapacitors was presented in Figure S4 [33]. At a scan rate of 50 mV s^−1^, the CV curve maintained a good rectangle when the voltage window is 0–1.2 V, indicating that the system showed negligible hydrogen evolution and oxygen evolution. When the voltage window increased to 1.3 V, the CV curve deviated from the rectangle at high potential. When the voltage window increased to 1.4 V and 1.6 V, the degree of deformation was further intensified. Specifically, the current increased significantly and oxygen evolution occurred obviously in the system at high voltage, so the voltage range was selected as 0–1.2 V.

In Figure S1a, the CV curve still maintains a nearly rectangular shape without obvious shift with the increasing scan rate, demonstrating that the hydrogel electrolyte provided a good conductive role in the gel, which was related to the electrolyte’s high conductivity. Figure S1b,d show that as the current density increased from 0.5 A g^−1^ to 20 A g^−1^, the curve still maintained a good triangular shape without obvious voltage drop, indicating the stability of the device at this voltage. The high ionic conductivity of the hydrogel electrolyte as well as its surface wrinkles and internal micropores promoted adsorption and desorption [34]. The CV curve in Figure S1c is similar to that in Figure S1a. However, the CV curve of the flexible device shifted slightly from the rectangle, and the current increased significantly when the scan rate and voltage window increased.

The EIS comparison plot of the two devices in Figure S2 validated the above conclusion. From the intersection and diameter of the semicircle and the abscissa in the high-frequency region, the ohmic impedance of the flexible hydrogel device is 0.40 Ω, which was slightly higher than that of the aqueous device (0.38 Ω) due to the slightly worse contact between the hydrogel electrolyte and the electrode. The electrochemical impedance of the flexible hydrogel device was 0.42 Ω, which was slightly larger than that of the aqueous device (0.25 Ω). The small electrochemical impedance presented the fast ion transfer speed in the hydrogel electrolyte due to its high ionic conductivity [35].

When the current density reached 0.5 A g^−1^, the specific capacitance of the flexible hydrogel device was 75.9 F g^−1^ (Figure S3a), which was lower than that of the aqueous device (98.8 F g^−1^) at the same current density due to the poor utilization of active materials and high Rct of flexible devices. Furthermore, when the current density increased to 20 A g^−1^, the specific capacitances of the gel and aqueous supercapacitors were 53.3 F g^−1^ and 80 F g^−1^, compared with the current density of 0.5 A g^−1^, which were reduced by 22.3 F g^−1^ and 18.8 F g^−1^, respectively. Figure S3b showed that the hydrogel supercapacitor has a higher energy density due to its wider voltage range. The highest energy density was 15.2 Wh kg^−1^. When the power density is increased to 12,000 W kg^−1^, the hydrogel device can still present an energy density of 10.7 Wh kg^−1^.

**Figure 7:**
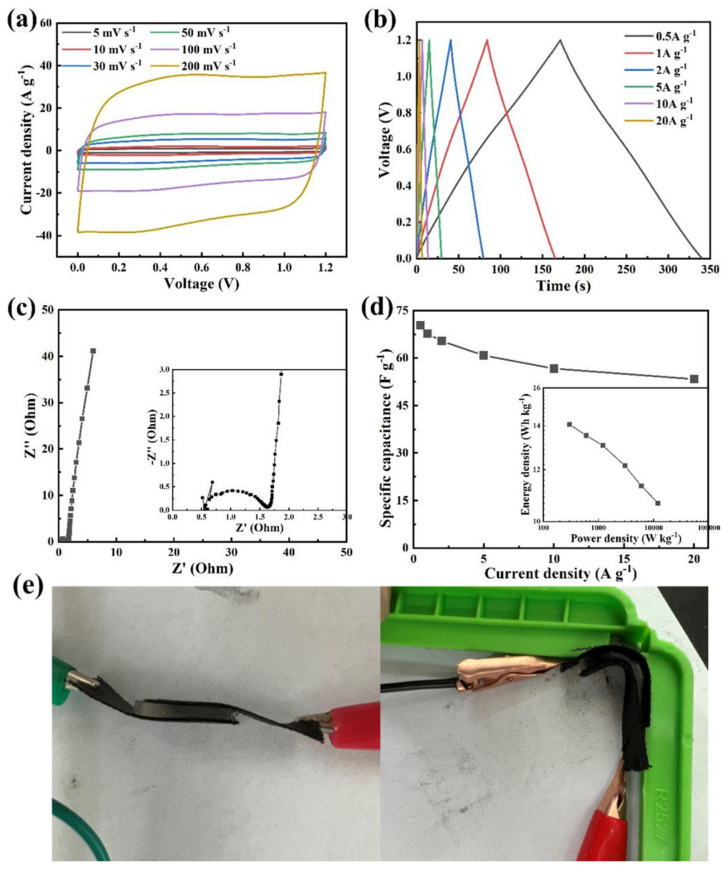
(**a**) CV curve, (**b**) GCD curve, (**c**) EIS curve; and (**d**) specific capacitance-current density curve (inset is power density-energy density curve) of hydrogel supercapacitor when bent at 90°. (**e**) Test digital photos when unbent and bent at 90°.

Flexible energy storage devices may be bent during daily use. We tested the flexible hydrogel supercapacitor by bending it at 90° (Figure 7a–d). The digital photos of the hydrogel supercapacitor during the test, are shown in Figure 7e. As shown in Figure 7a, the tested CV curve when bent at 90° still maintains good symmetry at 200 mV s^−1^, showing an approximately rectangular shape. As the current density increased from 0.5 A g^−1^ to 20 A g^−1^, the GCD curve showed a symmetrical triangle (Figure 7b), and there was a negligible shift with the increasing current density. When the density increased to 20 A g^−1^, it still maintained a small voltage drop due to the small impedance and large ionic conductivity of the flexible hydrogel supercapacitor.

The EIS curve in Figure 7c further confirmed this speculation. The ohmic impedance of the hydrogel supercapacitor (Figure S2a) bent at 90° was 0.55 Ω, which was larger than that before bending (0.4 Ω). Ion-electron transfer and conversion between gel electrolytes may be affected after bending [36]. In Figure 7d, the specific capacitance was 67.4 F g^−1^ at a low current density of 0.5 A g^−1^, which was 8.5 F g^−1^ lower than that before bending (Figure S3a). Correspondingly, its maximum energy density dropped to 13.5 Wh kg^−1^.

During practical applications, the flexible energy storage device should be bent many times [37]. We tested the electrochemical performance of the device after bending it 30 times to restore the original shape (Figure S5). In Figure S5a, even after bending for 30 times, the CV curve still maintained a good rectangle with a negligible shift at a high scan rate of 200 mV s^−1^, indicating its good mechanical stability. The GCD curve in Figure S5b showed a good symmetrical triangle, indicating good charge and discharge reversibility [38]. Moreover, there is no obvious voltage drop, demonstrating that the performance of the flexible hydrogel device was not affected after 30 times of bending damage. The EIS curve of Figure S5c showed that the ohmic impedance of the flexible hydrogel supercapacitor after 30 times of bending was low (0.62 Ω), indicating good contact between electrodes and the gel electrolyte with increasing bending times. Figure S5d showed that the supercapacitor after bending 30 times presented a specific capacitance of 70.3 F g^−1^ at 0.5 A g^−1^. As the current density increases to 20 A g^−1^, the supercapacitor showed a specific capacitance of 70.3 F g^−1^. It still had a high specific capacitance of 48.3 F g^−1^, which was only 5 F g^−1^ lower than that before damage, indicating that little damage to the supercapacitor after 30 times of bending.

**Figure 8:**
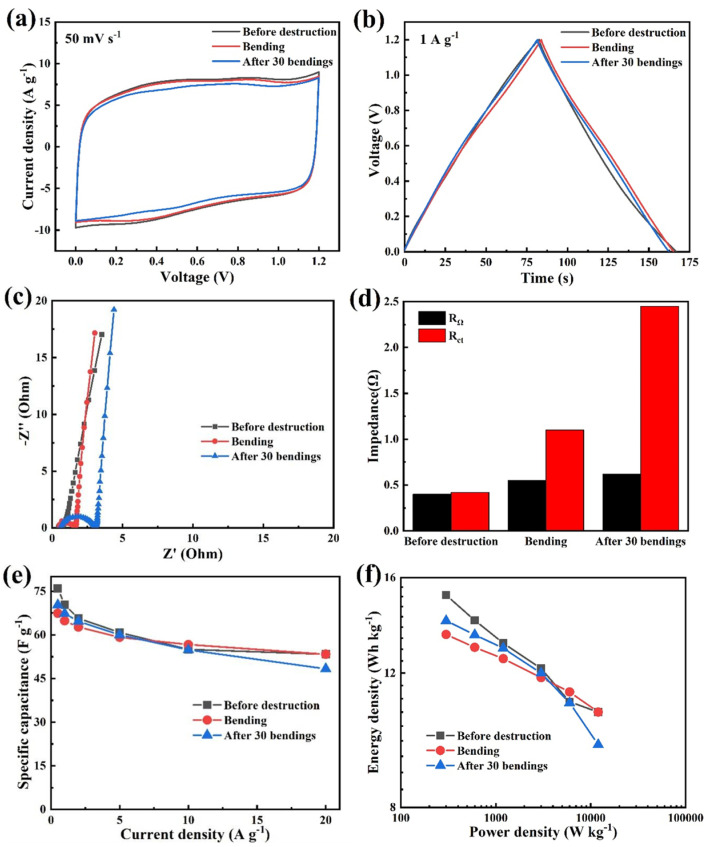
Performance comparisons of hydrogel supercapacitors in different states of (**a**) CV curve, (**b**) GCD curve, (**c**) EIS curve, (**d**) ohmic impedance and electrochemical impedance, (**e**) specific capacitance-current density curve, and (**f**) energy density-power density curve.

By comparing the performance of flexible hydrogel supercapacitors in different states, the performance of flexible hydrogel supercapacitors can be comprehensively evaluated. Figure 8a showed the CV curves of the hydrogel supercapacitor in the three states of undamaged, bent at 90°, and bent for 30 times at a scan rate of 50 mV s^−1^. These curves were all approximately rectangular and symmetrical, indicating that bending 90° and 30 times had a negligible effect on the hydrogel supercapacitor. In Figure 8b, at a current density of 1 A g^−1^, the triangles were symmetric with no obvious voltage drop, which was consistent with the high conductivity of the electrolyte. The flexible device has a small impedance, and the specific capacitances corresponding to the three states are 70.3 F g^−1^, 64.8 F g^−1,^ and 67.3 F g^−1^, respectively. The EIS curves are shown in Figure 8c–d. The slope of the straight line in the low-frequency region of the EIS image presents a fast electrolyte ion diffusion rate [39]. The smallest ohmic impedance before damage was 0.40 Ω (followed by 0.55 Ω when bent 90°) and the largest ohmic impedance after 30 times of bending is 0.62 Ω. The electrochemical impedance also shows similar characteristics. When bending at 90°, the electrochemical impedance was similar to that before bending, indicating that the bending state does not affect its electrochemical reaction [40]. When bending for 30 times, the ohmic impedance (2.45 Ω) was significantly larger than that before damage (0.42 Ω), indicating that the electrolyte had a slight change compared with the state before damage after repeated bending [41].

Figure 8e showed that the specific capacitance of the three states decreased with the increasing current densities from 0.5 A g^−1^ to 20 A g^−1^. When the current density was 0.5 A g^−1^, the undamaged supercapacitor has the largest specific capacitance of 75.9 F g^−1^. The specific capacitance of the supercapacitor in the state of recovery after 30 times of bending was 70.3 F g^−1^. The lowest specific capacitance in the 90° bending state was 67.4 F g^−1^. In the undamaged state, the specific capacitances of the supercapacitor in the three states drop to 48.3 F g^−1^, 53.3 F g^−1^, and 53.3 F g^−1^ at the current density of 20 A g^−1^, respectively. The Ragon plot curve of Figure 8f showed the same trend as the specific capacitance-current density. When the power density was 300 W kg^−1^, the device in the unbroken state reaches the highest energy density of 15.2 Wh kg^−1^. Moreover, the energy densities bending at 90° and for 30 times were 13.5 Wh kg^−1^ and 14.0 Wh kg^−1^, respectively. Finally, the cycle performance test of the flexible supercapacitor was carried out (Figure S6). At a high current density of 4 A g^−1^, the specific capacitance was 61.7 F g^−1^. After 10,000 cycles, the flexible supercapacitor showed a specific capacity of 44.8 F g^−1^ (with a capacity retention rate of 72.52%) and a coulombic efficiency of about 99.8%. These results indicated that the flexible supercapacitor has good energy conversion efficiency and reversibility.

## 4. Conclusions

In conclusion, PVA linked with PA showed high ionic conductivity, high transparency, and high tensile and compressive strengths. The introduction of phytic acid enables PVA and phytic acid to form intermolecular hydrogen bonds, providing new physical cross-linking points for PVA, thereby improving the formation conditions of PVA hydrogels. Phytic acid can ionize a large number of hydrogen ions in the hydrogel so that PVA-PA_21%_ without electrolyte and PVA-PA_21%_-2 M H_2_SO_4_ hydrogel presented ionic conductivity of 19.99 mS cm^−1^ and 62.75 mS cm^−1^. The supercapacitor of PVA-PA_21%_-2 M H_2_SO_4_ hydrogel showed a high specific capacitance of 70.3 F g^−1^ at the current density of 1 A g^−1^. While bending at 90° and for 30 times, the specific capacitances of the supercapacitors were still maintained at 64.8 F g^−1^ and 67.3 F g^−1^, respectively. The flexible hydrogel supercapacitor fabricated with the hydrogel electrolyte showed the highest energy density of 15.2 Wh kg^−1^ at the power density of 300 W kg^−1^. Moreover, the capacity retention rate was 72.52% after 10,000 charge-discharge cycles at a current density of 4 A g^−1^. These results demonstrated that the hydrogel electrolyte possesses practical application prospects.

## Figures and Tables

**Table 1:** The amount of different components added into PVA-PA_X_ gel samples.

Samples	PVA (g)	50 wt% PA (mL)	Deionized Water (mL)	PA (wt%)
PVA-PA_0%_	3	0	26.5	0
PVA-PA_7%_	3	3	22.3	7
PVA-PA_14%_	3	6	18.2	14
PVA-PA_21%_	3	9	14.1	21
PVA-PA_28%_	3	12	10.0	28
PVA-PA_35%_	3	15	5.8	35

## Data Availability

All data generated or analysed during this study are included in this published article (and its supplementary information files).

## Supplementary Materials

The following supporting information can be downloaded at: https://www.mdpi.com/article/10.3390/nano13030380/s1, Table S1. Summary of self-healing materials of this work compare to the other reports; Figure S1. (a) CV curve, (b) GCD curve of flexible hydrogel supercapacitor. (c) CV curves and (d) GCD curve of aqueous solution supercapacitor; Figure S2. (a) EIS comparison diagram of flexible hydrogel and aqueous solution supercapacitor. (b) Comparison diagram of Ohmic impedance and electrochemical impedance; Figure S3. (a) Specific capacitance-current density comparison diagram of flexible hydrogel and aqueous solution supercapacitors. (b) Power density-energy density comparison diagram; Figure S4. Determination of voltage window of PVA-PA_21%_-2 M H_2_SO_4_ flexible supercapacitor; Figure S5. (a) CV curve, (b) GCD curve, (c) EIS curve and (d) specific capacitance-current density curve (the inset is the power density-energy density curve) of hydrogel supercapacitor after bending for 30 times; Figure S6. Cyclic performance of flexible supercapacitors.
