# Supplementary material for: A Self-Healing PVA-Linked Phytic Acid Hydrogel-Based Electrolyte for High-Performance Flexible Supercapacitors
Source: Nanomaterials (Basel). 2023 Jan 17;13(3):380. doi: 10.3390/nano13030380 (PMC9920227; doi:10.3390/nano13030380)
Supplement: Supplementary file 1 [file nanomaterials-13-00380-s001.zip › nanomaterials-2115113-supplementary.pdf]

nanomaterials

A Self-Healing PVA-Linked Phytic Acid Hydrogel-Based Electrolyte for High-Performance Flexible Supercapacitors

Jing Zhao [1,2,†], Yuanqi Lu [1,†], Yuhua Liu [3], Lanxin Liu [1], Jinling Yin [1,*], Baozhi Sun [2], Guiling Wang [1] and Yongquan Zhang [1,*]

[1] Key Laboratory of Superlight Materials and Surface Technology of Ministry of Education, College of Materials Science and Chemical Engineering, Harbin Engineering University, Harbin 150001, China; zhtdzhn@163.com (J.Z.); luyuanqi@hrbeu.edu.cn (Y.L.); liulanxin@hrbeu.edu.cn (L.L.); wangguiling@hrbeu.edu.cn (G.W.)

[2] College of Power and Energy Engineering, Harbin Engineering University, Harbin 150001, China; sunbaozhi@hrbeu.edu.cn

[3] Jixi Quality Inspection and Testing Center of Graphite Product, Jixi 158100, Heilongjiang, China; huahua9700@163.com

* Correspondence: yinjinling@hrbeu.edu.cn (J.Y.); yqzhang@hrbust.edu.cn (Y.Z.)

† These authors contributed equally to this work.

Table S1. Summary of self-healing materials of this work compare to the other reports.

**Materials**	**PVA/PA/Fe^{3+} (PPFe) hydrogel**	**PVA–PA gels**	**GelMA-CNC hydrogel**	**PVA-TA-H_3PO_4 GPE**	**CNT@graphene@PANI-1 film**	**stand-alone carbyne (SAC) film**	**Graphite/bPEI**	**(PVA)/H_3PO_4 gel**	**PVA-$PA_{21\%}$ hydrogel**
Tensile stress	0.618 MPa	N/A	17.42 KPa	1.19 MPa	N/A	N/A	6 MPa	N/A	805 kPa
Tensile strain	924 %	1100%	63%	N/A	180%	N/A	35	N/A	276.5%
Energy density	0.212 mWh/cm^2	N/A	20.65 mWh cm^{-3}	N/A	36.3 μW h cm^{-2}	703.25 μF V_2 cm^{-2}	N/A	5.8 μWh cm^{-2}	13.5 Wh kg^{-1}
Ref	[1]	[2]	[3]	[4]	[5]	[6]	[7]	[8]	This work

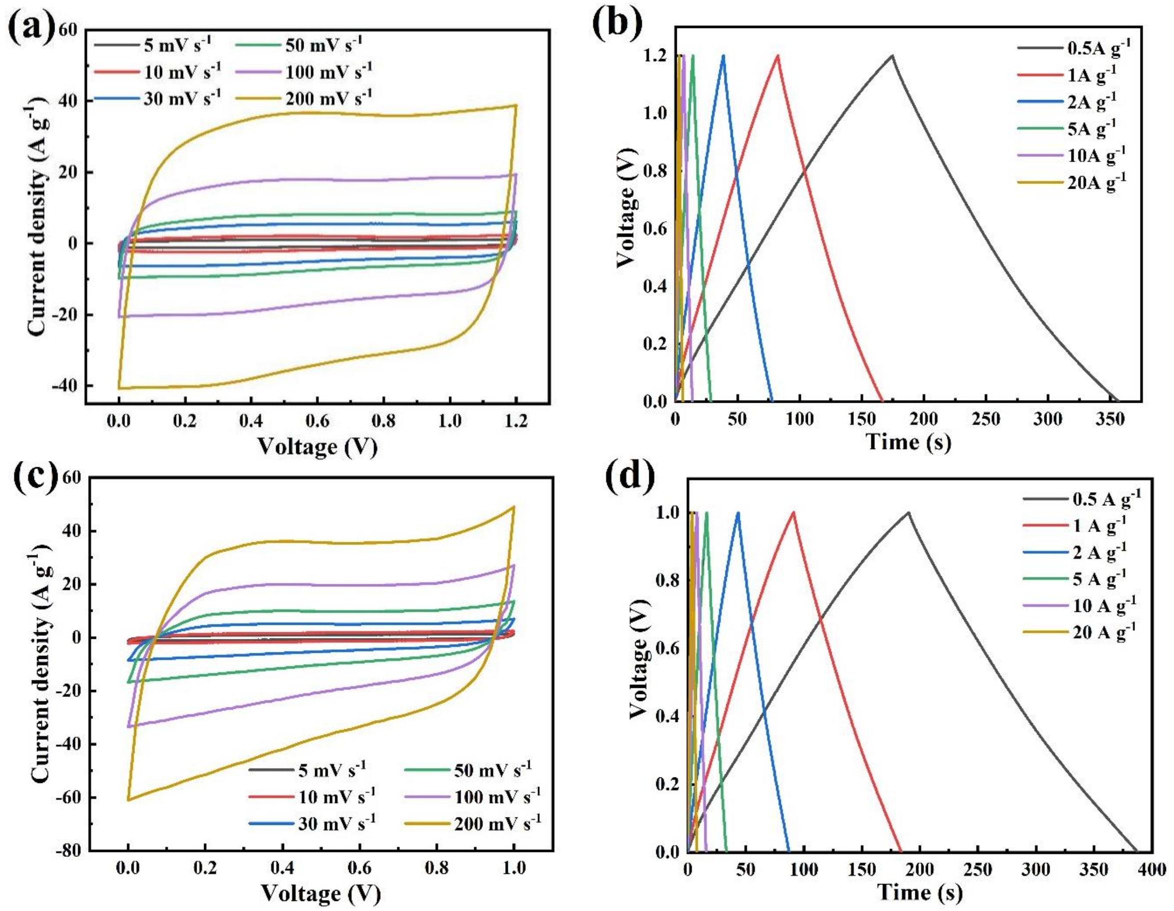

Figure S1. (**a**) CV curve, (**b**) GCD curve of flexible hydrogel supercapacitor. (c) CV curves and (d) GCD curve of aqueous solution supercapacitor.

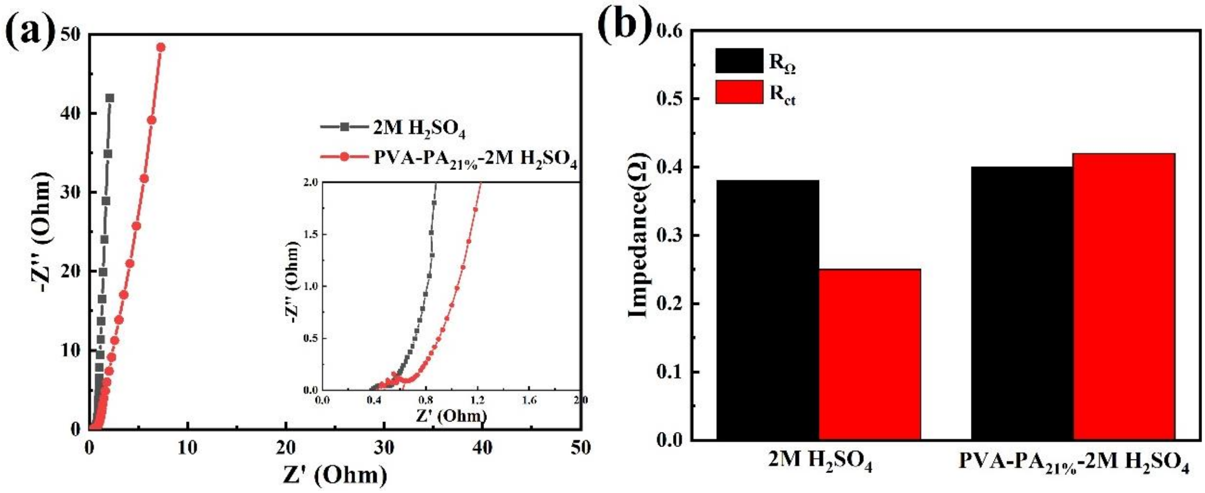

Figure S2. (**a**) EIS comparison diagram of flexible hydrogel and aqueous solution supercapacitor. (**b**) Comparison diagram of Ohmic impedance and electrochemical impedance.

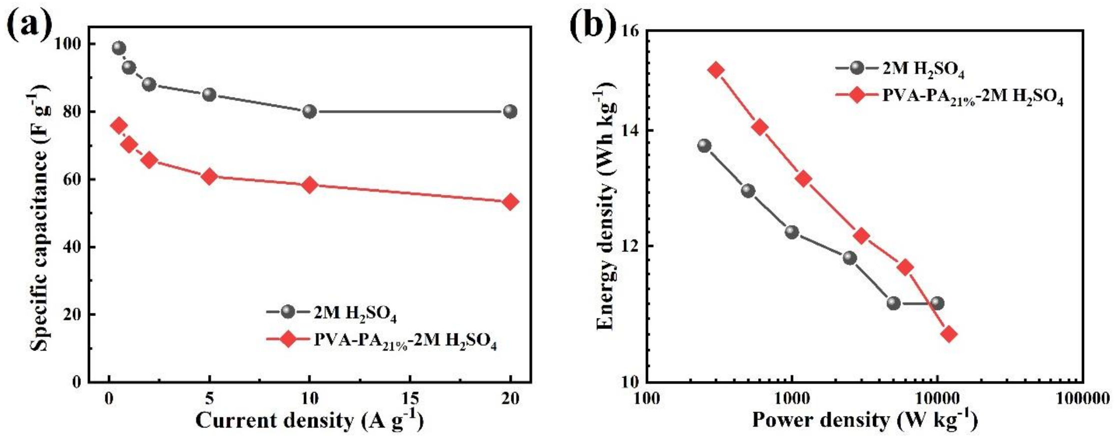

Figure S3. (**a**) Specific capacitance-current density comparison diagram of flexible hydrogel and aqueous solution supercapacitors. (**b**) Power density-energy density comparison diagram.

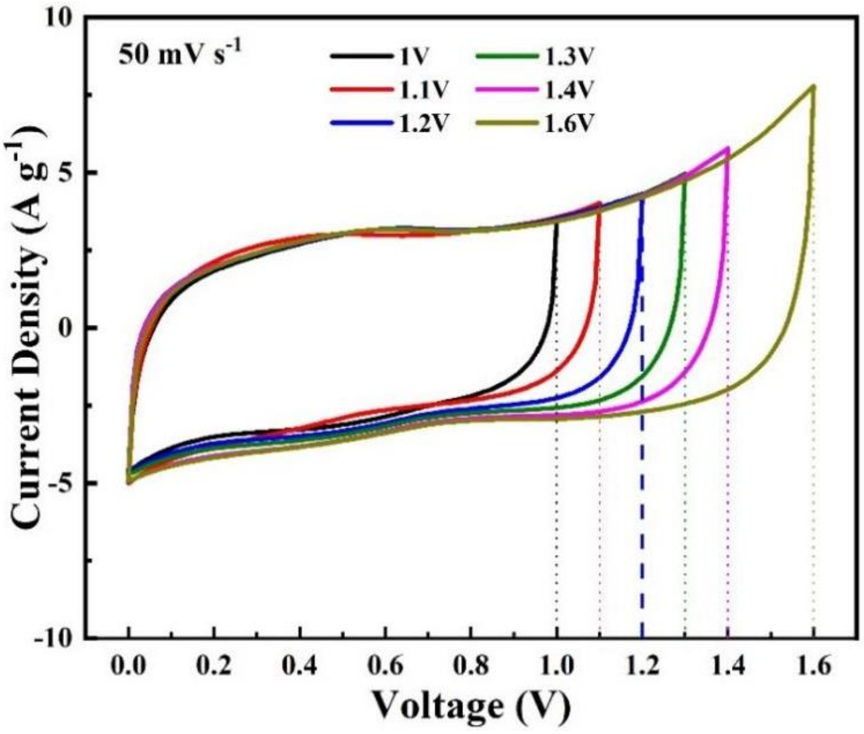

Figure S4. Determination of voltage window of PVA-$PA_{21\%}$-2 M H_2SO_4 flexible supercapacitor.

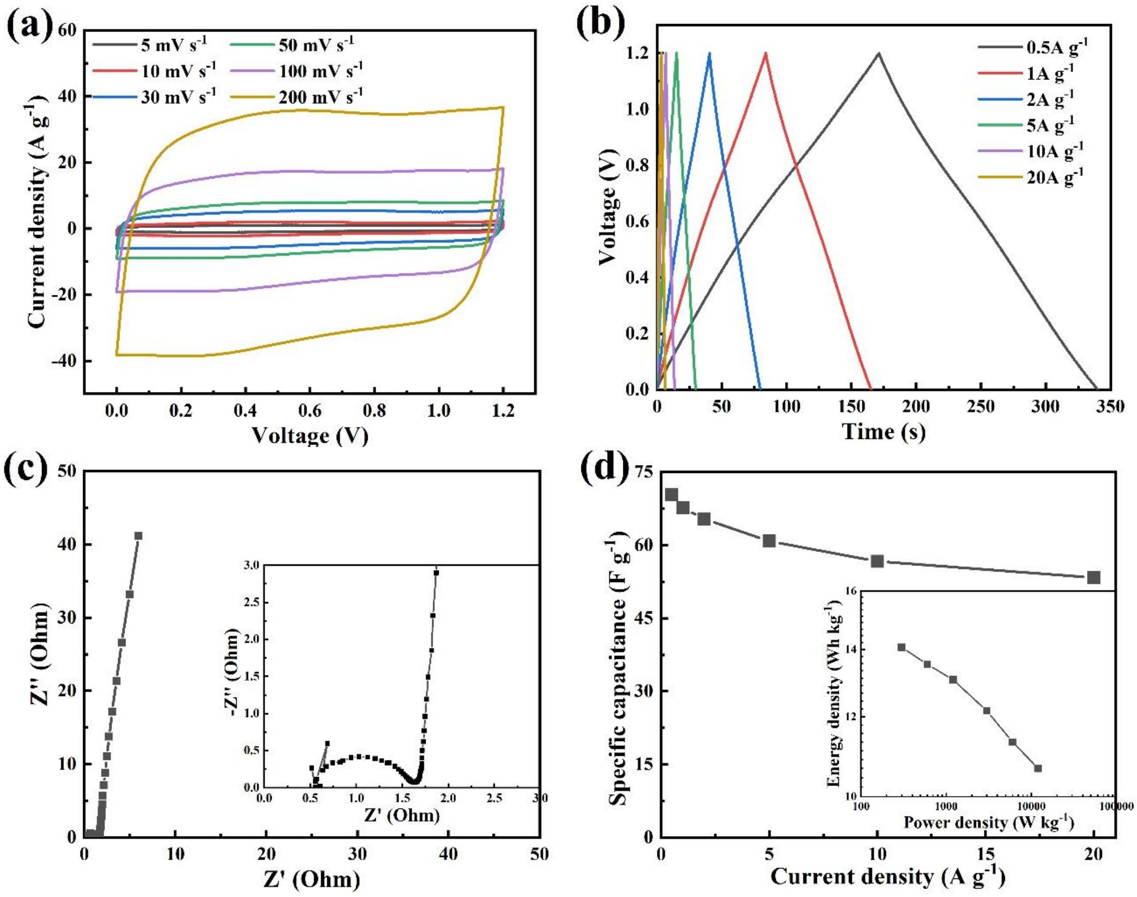

Figure S5. (**a**) CV curve, (**b**) GCD curve, (**c**) EIS curve and (**d**) specific capacitance-current density curve (the inset is the power density-energy density curve) of hydrogel supercapacitor after bending for 30 times.

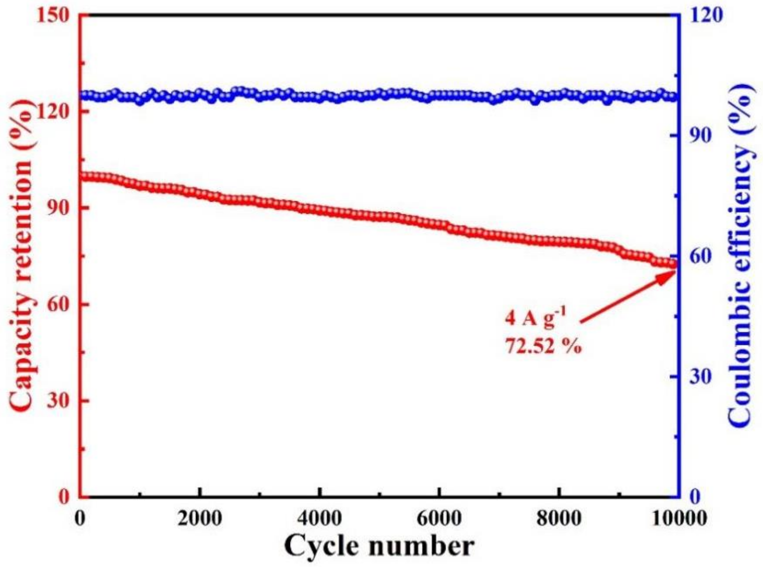

Figure S6. Cyclic performance of flexible supercapacitors.

References

1. Lin, J.-h.; Du, X.-s. Self-healable and redox active hydrogel obtained via incorporation of ferric ion for supercapacitor applications. *Chemical Engineering Journal* **2022**, *446*.
2. Zhang, S.;Zhang, Y.;Li, B.;Zhang, P.;Kan, L.;Wang, G.;Wei, H.;Zhang, X.; Ma, N. One-Step Preparation of a Highly Stretchable, Conductive, and Transparent Poly(vinyl alcohol)-Phytic Acid Hydrogel for Casual Writing Circuits. *ACS Appl Mater*

Interfaces **2019**, *11*, 32441-32448.

3. Hsu, H. H.;Liu, Y.;Wang, Y.;Li, B.;Luo, G.;Xing, M.; Zhong, W. Mussel-Inspired Autonomously Self-Healable All-in-One Supercapacitor with Biocompatible Hydrogel. *ACS Sustainable Chemistry & Engineering* **2020**, *8*, 6935-6948.
4. Qin, G.;Wang, M.;Fan, L.;Fang, X.;Zhang, D.;Liu, J.;Qin, J.;Shi, J.;Yang, J.; Chen, Q. Multifunctional supramolecular gel polymer electrolyte for self-healable and cold-resistant supercapacitor. *Journal of Power Sources* **2020**, *474*.
5. Liang, X.; Zhao, L.;Wang, Q.;Ma, Y.; Zhang, D. A dynamic stretchable and self-healable supercapacitor with a CNT/graphene/PANI composite film. *Nanoscale* **2018**, *10*, 22329-22334.
6. Mariappan, V. K.;Krishnamoorthy, K.;Manoharan, S.;Pazhamalai, P.; Kim, S. J. Electrospun Polymer-Derived Carbyne Supercapacitor for Alternating Current Line Filtering. *Small* **2021**, *17*, e2102971.
7. Wu, T.; Chen, B. A mechanically and electrically self-healing graphite composite dough for stencil-printable stretchable conductors. *Journal of Materials Chemistry C* **2016**, *4*, 4150-4154.
8. Vu, V.-P.;Mai, V.-D.;Nguyen, D. C. T.; Lee, S.-H. Flexible and Self-Healable Supercapacitor with High Capacitance Restoration. *ACS Applied Energy Materials* **2022**, *5*, 2211-2220.
